# Pathological complete response after nivolumab therapy following angiogenesis inhibitors in a patient with metastatic renal cell carcinoma

**DOI:** 10.1002/iju5.12220

**Published:** 2020-10-05

**Authors:** Hiroki Hagimoto, Soki Kashima, Kazuki Doi, Shintaro Nakayama, Takanori Sano, Satoshi Imai, Tomihiko Yasufuku, Mototsugu Muramaki, Yuji Yamada

**Affiliations:** ^1^ Department of Urology Hyogo Prefectural Amagasaki General Medical Center Amagasaki Hyogo Japan; ^2^ Department of Urology Kyoto University Graduate School of Medicine Kyoto Kyoto Japan; ^3^ Department of Urology Akita University Graduate School of Medicine Akita Akita Japan

**Keywords:** metastasectomy, nivolumab, renal cell cancer

## Abstract

**Introduction:**

Nivolumab is effective for advanced renal cell carcinoma; however, reports are limited wherein nivolumab is combined with sequential therapy of angiogenesis inhibitors and metastasectomy.

**Case presentation:**

A 65‐year‐old man was diagnosed with left renal cell carcinoma of cT2aN0M1 with lung metastasis. The patient underwent nephrectomy and sequential therapy with interferon‐α and angiogenesis inhibitors. Lung metastasis decreased by angiogenesis inhibitors, but new right adrenal gland metastasis appeared. Nivolumab as the fifth systemic therapy remarkably shrank the metastasis. After discontinuing nivolumab therapy, the metastasis continued to shrink. The patient underwent adrenalectomy, and pathological analysis revealed no remnant cancer cells in the specimen, confirming a pathological complete response. Twenty months postoperatively, he remains in good health without recurrence.

**Conclusion:**

We report a rare case with renal cell carcinoma of a pathological complete response by nivolumab after angiogenesis inhibitors.

**Abbreviations & Acronyms**
CT: computed tomography
ICI: immune checkpoint inhibitor
IFNα: interferon‐α
IMDC: International mRCC Database Consortium
irAE: immune‐related adverse event
pCR: pathological complete response
PD‐1: programmed cell death 1
PD‐L1: programmed cell death ligand 1
RCC: renal cell carcinoma
TIL: tumor‐infiltrating lymphocyte
TKI: tyrosine kinase inhibitor
Treg: regulatory T cells

**Keynote message**

We report a metastatic RCC patient with continued response after nivolumab therapy following angiogenesis inhibitors. Pathological analysis of resected metastasis revealed a high density of tumor‐infiltrating CD8^+^ T cells and no viable cells after nivolumab.

## Introduction

In 2015, the U.S. Food and Drug Administration approved nivolumab therapy as a second‐line treatment for patients with advanced RCC.^1^, ^2^ Here we report a case of pCR with metastasectomy after nivolumab therapy following angiogenesis inhibitors.

## Case presentation

**Fig. 1:**
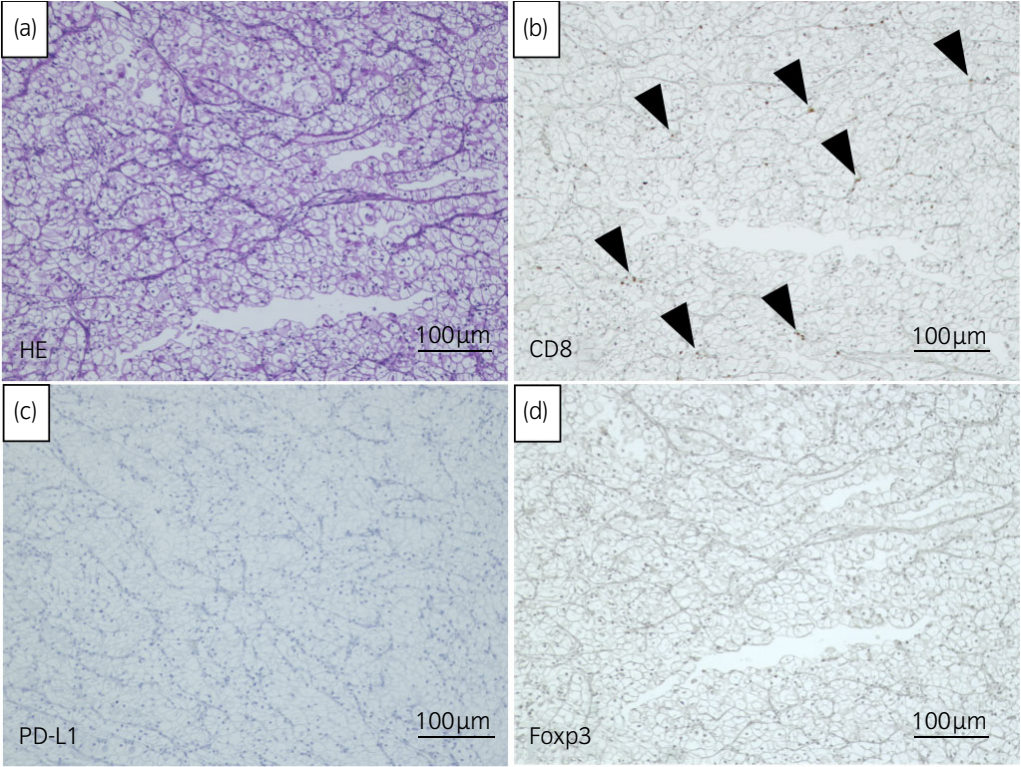
Immunohistochemical staining of primary renal tumor. (a) Hematoxylin‐eosin staining. (b) CD8^+^ staining. Black arrowheads indicate CD8^+^ T cells. (c) PD‐L1 staining. (d) Foxp3 staining.

A 65‐year‐old man with anemia and general malaise was referred to our hospital. CT imaging revealed 8 × 8 cm left renal tumor and multiple lung metastases. RCC of cT2aN0M1 was diagnosed, with intermediate and poor risks in the Memorial Sloan Kettering Cancer Center and IMDC risk scores, respectively. Left nephrectomy was performed, and pathological findings showed clear cell RCC, pT3b (Fig. 1a). Immunohistochemical analysis revealed infiltrating CD8^+^ TILs (Fig. 1b), no PD‐L1 expression (Fig. 1c), and Foxp3 + Treg as the suppressive factors in tumor immunity (Fig. 1d).

IFNα shrank lung metastasis resulting in 72% decrease consistent with partial response (RECIST version 1.1). As lung metastasis had not enlarged, IFNα was discontinued 5 years postoperatively.

**Fig. 2:**
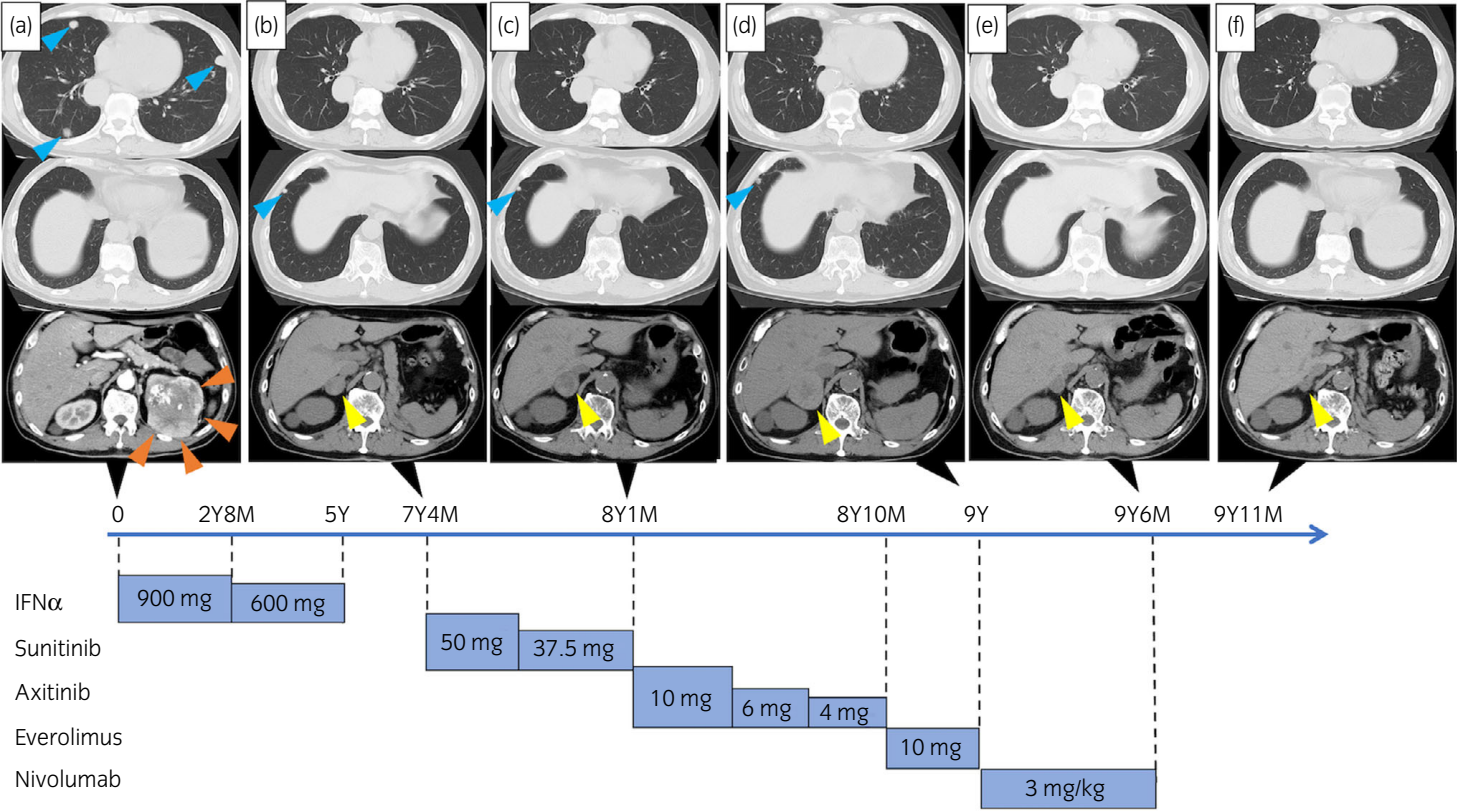
Clinical course of the case. CT images of representative lesions of the renal tumor (orange arrowheads), lung metastasis (blue arrowheads), and right adrenal gland metastasis (yellow arrowheads). (a) CT scan at primary diagnosis. (b) CT revealed new lung and right adrenal gland metastasis. (c) CT revealed lung metastasis shrinkage but adrenal gland metastasis growth. (d) CT revealed adrenal gland metastasis growth. (e) CT revealed adrenal gland metastasis remarkably reduced in size by nivolumab. (f) Five months after discontinuing nivolumab, CT revealed that adrenal gland metastasis continued to shrink. IFNα was administered weekly, each of the TKIs daily, and nivolumab once every 2 weeks.

New lung and adrenal gland metastases appeared 7 years and 4 months after nephrectomy. Following sunitinib treatment, CT revealed shrinking lung metastasis but growing right adrenal metastasis (Fig. 2).

Axitinib therapy commenced and after 9 months, CT revealed 58% increase in adrenal metastasis growth.

Everolimus was initiated as a fourth‐line therapy, but 2 months later it was discontinued due to grade 1 acute kidney injury (Common Terminology Criteria for Adverse Events version 5.0).

The patient complained of fatigue, and laboratory tests revealed high C‐reactive protein and low hemoglobin levels due to progression.

Nivolumab was administered as the fifth‐line therapy; thereafter, the patient’s symptoms and laboratory results dramatically improved. Lung metastasis diminished with complete response, and grade 2 maculopapular rash appeared. By the seventh course, grade 2 acute kidney injury developed, which was clinically diagnosed as irAE. At the thirteenth course, nivolumab therapy was discontinued due to irAE. CT revealed 59% decrease in adrenal metastasis since starting nivolumab. After administering oral prednisolone (20 mg/day), the rash and renal dysfunction improved.

**Fig. 3:**
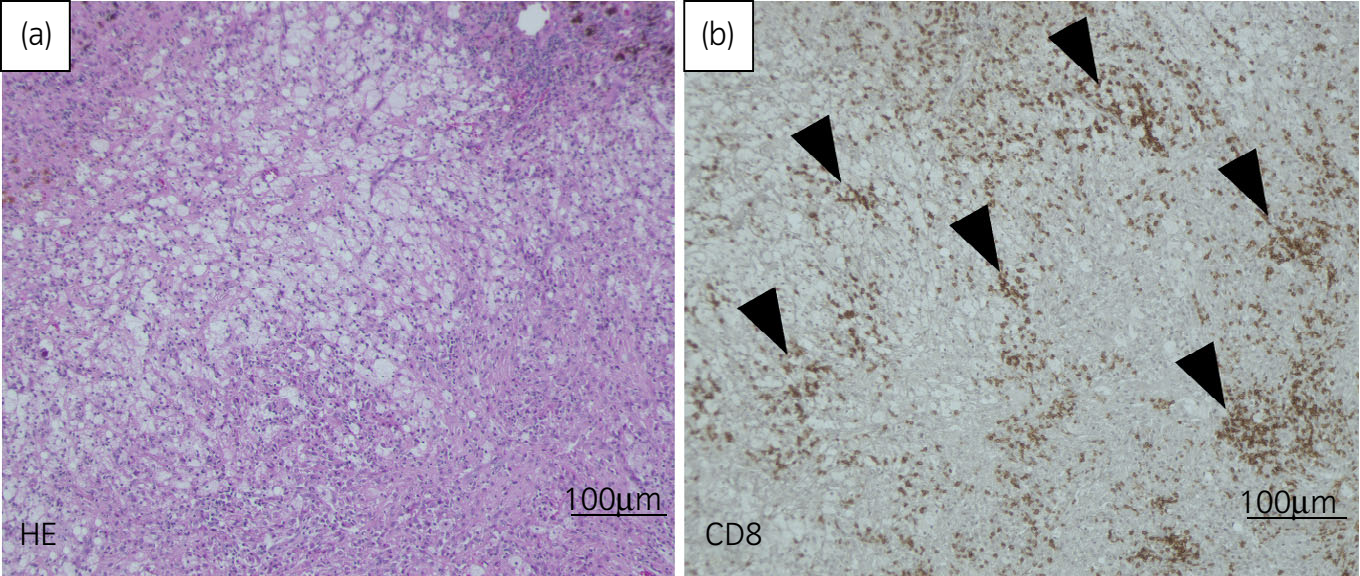
Immunohistochemical staining of resected adrenal gland metastasis. (a) Hematoxylin‐eosin staining. No viable cells were observed. (b) CD8 staining. CD8^+^ T cells accumulated in the adrenal glands.

After that, the metastasis continued to shrink resulting in further 11% decrease. Given the possibility of existing viable cells and the limited options of further systemic treatment for this case, we performed adrenalectomy to obtain surgical complete response. Pathological findings showed no viable cells in the surgical specimen (Fig. 3a). CD8^+^ TILs were remarkably observed in the metastasis treated with nivolumab (Fig. 3b). No local recurrence or metastasis has been observed for >20 months postoperatively.

## Discussion

Nivolumab shows antitumor effects by inhibiting PD‐1/PD‐L1 interaction and activating T cells in various cancer types.^3^ In the Checkmate 025 trial, the objective response rate was greater with nivolumab than with everolimus for previously treated patients with advanced RCC.^4^ However, nivolumab has therapeutic effects only in some RCC patients.^4^ Therefore, further research on ICI biomarker use in RCC is necessary to better understand their effects.

PD‐L1 expression in tumors is related to the efficacy of nivolumab monotherapy in melanoma and non‐small‐cell lung cancer;^5^ however, not related to efficacy by nivolumab in RCC.^3^ It is controversial what PD‐L1 expression means except in some carcinomas.^6^ In our case, PD‐L1 was not expressed in the primary renal tumor (Fig. 1c) and was never examined in the adrenal metastasis before treatment with nivolumab. Therefore, we are unable to discuss the relationship between the expression of PD‐L1 and the therapeutic effect of nivolumab in our case.

The CD8^+^/Treg balance in our case may suggest that this tumor is an immune‐activated tumor.^7^ Pre‐existing CD8^+^ T cells located at the tumor margin may predict response to pembrolizumab in patients with melanoma.^3^ A high density of intratumoral CD8^+^ T cells is associated with favorable cancer‐specific survival in RCC patients.^8^ In our case, the high density of CD8^+^ TILs in the metastasis probably resulted from a proliferation of T cells caused by ICI (Fig. 3b).

Including the present case, five cases of RCC that achieved pCR by nivolumab administration have been reported (Table 1).^9^, ^10^, ^11^, ^12^ The features that these cases have in common are: (i) all cases were treated with nivolumab following with TKI, and (ii) although the tumor mass shrank by nivolumab and remained on CT, there were no viable cells in the resected tumor. Regarding (i), recent studies have revealed the effectiveness of combination therapies by TKI and ICI as first‐line treatments for advanced RCC.^13^, ^14^ Vascular endothelial growth factor‐targeted treatment suppresses angiogenesis and reprograms the immune microenvironment.^15^ In our case, sequential angiogenesis inhibitor therapies may have changed the microenvironment resulting in the effectiveness of nivolumab treatment. Regarding (ii), ICI works remarkably well in certain cases. It has been suggested that pCR is not rare in cases with radiological partial response because not all undergo resection.^10^, ^11^, ^12^ In the present case, we had the option to follow up without surgery. However, cases in which viable cells exist have been reported after a radiographic response was obtained by ICI,^16^, ^17^ and we cannot predict whether shrunken lesions include viable cells. Therefore, the important roles of metastasectomy in this TKI and ICI era are to obtain surgical complete response, confirm pathology, and discontinue ICI. Furthermore, it was also reported that complete metastasectomy of RCC might be associated with improved overall survival in the era of targeted therapy and ICI.^18^ Referring to the above‐mentioned present discussion, if all metastases remain shrunken and resectable after nivolumab therapy, metastasectomy may be performed to obtain surgical complete response depending on the individual’s situation.

**Table 1:** Summary of previously reported cases of metastatic RCC with pathologically complete response after treatment with nivolumab

Author (year)	Age (years)	Sex	cStage (metastatis)	Side of RCC	Pathology	IMDC	Resected region	Nivolumab dose term	irAE	Treatments before ICI	Disease‐free survival
Ikarashi *et al*.^9^ (2018)	68	Female	T4N0M1 (lung, liver)	Right	Clear cell	Poor	Kidney, liver	3 mg/kg 16 courses	Uveitis	Sunitinib	3 months
Shirotake *et al*.^10^ (2019)	52	Male	T1bN1M1 (brain, lung)	Left	Clear cell	N/A	Kidney	3 mg/kg 5 courses	–	Pazopanib, everolimus, axitinib, radiotherapy	4 months
Bhat *et al*.^11^ (2019)	57	Female	T4N1M1 (right atrium, liver)	Right	Clear cell	N/A	Kidney	N/A	–	Pazopanib, cabozantinib	N/A
Shiraishi *et al*.^12^ (2019)	N/A	N/A	T3cN2M0	Right	N/A	N/A	Kidney	3 mg/kg 18 courses	–	Pazopanib	N/A
Present case	65	Male	T2aN0M1 (lung, adrenal gland)	Right	Clear cell	Poor	Adrenal gland	3 mg/kg 16 cources	Interstial nephritis, skin rash	IFN, sunitinib, axitinib, everolimus	20 months

## Conclusion

A patient with metastatic RCC was treated with nivolumab therapy following angiogenesis inhibitors. All metastatic lesions were remarkably reduced in size. Adrenal metastases continued to shrink after nivolumab therapy was discontinued. The resected adrenal lesion did not include any viable cells, resulting in pCR. The patient has been healthy without recurrence for >20 months.

## Study approval

This study was approved by the institutional review board of Hyogo Prefectural Amagasaki General Medical Center and was conducted in accordance with the tenets of the Declaration of Helsinki. All specimens were collected from the patient after written informed consent was obtained.

## Conflict of interest

The authors declare no conflict of interest.
